# Insight into the function of tetranectin in human diseases: A review and prospects for tetranectin-targeted disease treatment

**DOI:** 10.1016/j.heliyon.2023.e23512

**Published:** 2023-12-10

**Authors:** Sana Iram, Safikur Rahman, Inho Choi, Jihoe Kim

**Affiliations:** a Department of Medical Biotechnology and Research Institute of Cell Culture, Yeungnam University, Gyeongsan, 38541, Republic of Korea; b Department of Botany, Munshi Singh College, BR Ambedkar Bihar University, Muzaffarpur, Bihar, 845401, India

**Keywords:** Tetranectin, Cancers, Developmental disorders, Neurological diseases, Inflammation, Diabetes

## Abstract

Tetranectin (TN), a serum protein, is closely associated with different types of cancers. TN binds plasminogen and promotes the proteolytic activation of plasminogen into plasmin, which suggests that TN is involved in remodeling the extracellular matrix and cancer tissues during cancer development. TN is also associated with other diseases, such as developmental disorders, cardiovascular diseases, neurological diseases, inflammation, and diabetes. Although the functional mechanism of TN in diseases is not fully elucidated, TN binds different proteins, such as structural protein, a growth factor, and a transcription regulator. Moreover, TN changes and regulates protein functions, indicating that TN-binding proteins mediate the association between TN and diseases. This review summarizes the current knowledge of TN-associated diseases and TN functions with TN-binding proteins in different diseases. In addition, potential TN-targeted disease treatment by inhibiting the interaction between TN and its binding proteins is discussed.

## 1 Introduction

Tetranectin (TN) was first discovered in 1986 in human serum as a plasminogen-binding protein [1]. TN promotes the proteolytic activation of plasminogen into plasmin with increasing fibrinolysis. The serum TN concentrations in healthy individuals are ∼10 mg/L, whereas cancer patients showed significantly lower serum TN levels [[2], [3], [4], [5], [6], [7], [8]] (Table 1). These reports indicated the close association between TN and cancers, and suggested TN as a serum biomarker. TN has been extensively investigated to elucidate the functional mechanism of TN in cancer development. It was revealed that TN accumulates in the extracellular matrix (ECM) in cancer tissues and co-localizes with plasminogen in an invasive front [[9], [10], [11]]. This finding, together with the molecular function of TN in plasminogen activation, suggests the involvement of TN in cancer development by tissue remodeling.

**Table 1:** Human diseases associated with TN.

Diseases		Correlation with TN levels	Potential Mediators	References
Cancers	Colorectal Cancer	Serum TN ↓ (8.96 mg/L) Plasma TN ↓ (<7.5 mg/L)	Plasminogen	[2,3]
	Metastatic Breast Cancer	Serum TN ↓ (8.7 mg/L)	Plasminogen	[5]
	Multiple Myeloma	Serum TN ↓ (8.2–9.2 mg/L)	Plasminogen	[7,8]
	Ovarian cancer	Plasma TN ↓ (6.7 mg/L) Stromal TN ↑	Plasminogen	[22]
	Cutaneous melanoma Lesions	Stromal TN ↑	Plasminogen∗	[25]
	Metastatic Oral Cancer	Saliva and serum TN expression ↓	Plasminogen	[26]
	Bladder Cancer	Stromal TN ↑	Plasminogen	[27]
	Gastric Cancer	Intratumoral TN expression ↑	Plasminogen	[28]
	Clear Cell Renal Cell Carcinoma	TN gene and protein expression ↓ Therapeutic target	Unknown	[29]
	Lung Cancer	TN gene expression ↓	Plasminogen	[31]
	Hepatocellular Carcinoma	TN gene expression ↓	Unknown	[32]
Neurological disorders	Multiple Sclerosis	CSF TN ↓ (0.29 mg/L)	Unknown	[47]
	Epilepsy	Serum TN ↓ (6.7 mg/L) Plasma TN ↓ (6.2–7.5 mg/L) CSF TN ↑ (0.368 mg/L)	Plasminogen	[48,49]
	Parkinson’s Disease	CSF TN ↓ Caused in TN gene knock out mice	Plasminogen∗	[52,53]
Cardiovascular diseases	Heart Failure	Serum TN ↓ (30 ng/mL)	Unknown	[40]
	Acute Coronary Artery Disease	Serum TN ↓ (9.30–10.12 mg/mL)	Unknown	[61]
	Acute Myocardial Infarction	Plasma TN ↓ (8.27 mg/L)	Unknown	[63]
Other diseases	Kyphosis	Caused in TN gene knock out mice	Unknown	[14]
	Sepsis	Serum TN ↓ (3–4 μg/mL) Therapeutic target	HMGB1∗	[19]
	Type 2 Diabetes and Obesity	Serum TN ↑ (12–13 μg/mL) Therapeutic target	Ca^2+^/Ca^2+^ channel auxillary protein	[77]

∗ proved by cellular and molecular experiments.

TN also plays an important role in developmental disorders. TN expression increases significantly during the mineralization stage of bone development [12,13]. The disruption of TN expression led to kyphosis a kind of spinal cord deformity in a mouse model [14]. Moreover, TN expression increased significantly during mouse embryonic development and limb formation [15]. High TN expression was also observed in the muscle regeneration of adult mice. Recently, TN was also identified as an adipogenic serum protein that enhances adipogenesis [16,17]. The adipogenic function of TN was mediated by enhancing mitotic clonal expansion in the early phases of adipogenesis [18]. These reports indicate that TN regulates stem cell differentiation, but the molecular mechanism is not completely understood.

**Fig. 1:**
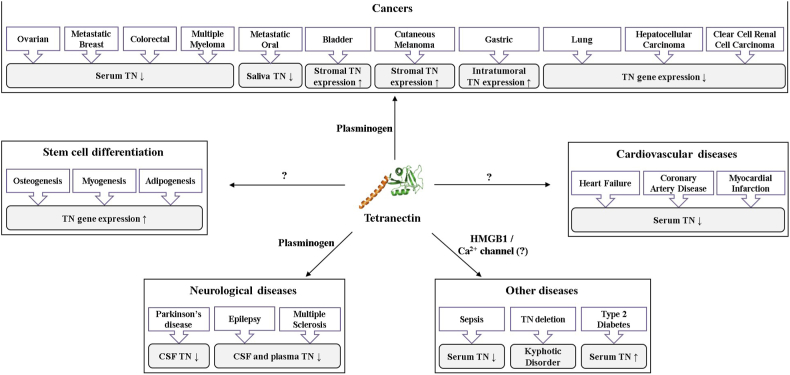
Human diseases associated with TN. TN-associated diseases reported in the literature are summarized with changes in TN levels (determined values are in Table 1). The TN-binding proteins with arrows indicate the potential mediators of TN functions in different diseases, and question marks are for unknown mediators.

In addition to cancers and developmental diseases, TN is associated with other diseases, including neurodegeneration, atherosclerosis, cardiovascular disease, diabetes, sepsis, and inflammation (Fig. 1 and Table 1). On the other hand, there is still a huge lack of research in terms of understanding the functions and mechanistic role of TN in different diseases. Other than plasminogen, TN binds many proteins with different roles, including structural proteins, growth factors, and transcription regulators (Table 2). These protein functions appeared to be changed and regulated by forming a complex with TN, which can be a diseases-causing mechanism. Recent studies reported that the inhibition of TN binding to the target proteins, HMGB1 and plasminogen, are effective to suppress lethal sepsis and colon cancer proliferation, respectively [19,20]. These studies strongly indicate that TN can be a drug target to treat various diseases by inhibiting the complex formation between TN and different binding proteins. In this regard, we summarized TN-associated human diseases, and potential or elucidated TN functions with binding molecules, which have been reported in the literature by searching databases (mainly PubMed, Web of Science, and Google Scholar). Moreover, we discussed development of TN-targeted inhibitors to treat different diseases.

**Table 2:** TN-binding proteins and molecules.

Binding molecules	Molecular structures (PDB ID)	Molecular functions	TN binding domain	Binding affinity	References
Plasminogen		Protease	Kringle 4 binding domain	*K*_d_ = 20–30 μM	[88,89,94]
Fibrin		Structural protein	ND	25 nmol/L*	[91,95]
Apolipoprotein A		Structural protein	Kringle 4 binding domain	*K*_d_ = 0.013 μM	[90,97]
Hepatocyte growth factor		Growth hormone	Kringle 4 binding domain	*K*_d_ = 0.49 μM	[99,100]
Plasminogen activator		Protease	Kringle 4 binding domain	*K*_d_ = 0.29 μM	[100]
High mobility group box-1		Transcription regulator	Kringle 4 binding domain	*K*_d_ = 1.21–2.88 nM	[19]
Heparin		Anticoagulant	Heparin-binding domain	*K*_d_ = 65 ± 2.0 μM	[82]
Epigallocatechin gallate		Natural compound	Kringle 4 binding domain	*K*_d_ = 3–6 μM	[20]
Diosgenin		Natural compound	Kringle 4 binding domain	ND	[114]

ND, unknown; *, amount of TN determined in clot lysates, which correspond to 13–17 % of plasma TN.

## 2 TN-associated human diseases

### 2.1 Cancers

An early study reported that serum TN levels were ∼10 mg/L in healthy individuals, with variations depending on age and sex [21]. In contrast, the serum TN levels were reduced significantly to ∼7 mg/L in patients with various cancers. Moreover, metastatic cancer patients showed lower serum TN levels compared with non-metastatic cancer patients, indicating the important role of TN in cancer development and progression [6]. Investigations of different types of cancers suggested TN as a prognostic and diagnostic cancer marker (Fig. 1 and Table 1). The study of breast cancer reported low serum TN levels of ∼8.4 mg/L in metastatic patients [5]. In addition, the serum TN levels followed after chemotherapy showed a significant negative correlation with patient survival. Despite the statistical variations between different types of cancers, most studies showed similar results that TN could be a biomarker for the development and progression of ovarian and colorectal cancers [3,4,22]. Histological analysis showed the deposition of TN in the ECM of cancer cells, whereas TN was not detected in the ECM of normal cells [9,[22], [23], [24]]. These results suggested that TN plays a role in ECM. Although the role of TN is unclear, it was suggested that the TN activity of enhancing plasminogen activation is responsible for cancer development by remodeling cancer tissues. This hypothesis is supported by a study on cutaneous melanoma lesions showing co-localization of TN with plasminogen/plasmin in the invasive front of melanomas (Table 1) [25]. TN accumulated in ECM is supposedly derived from the blood because the serum TN levels are reduced significantly in cancer patients. Alternatively, some cancer cells or cells associated with cancer tissues can express TN and secrete it into the ECM. On the other hand, TN expression in cancer tissues was correlated inconsistently with cancer development and metastasis in different types of cancers [23,24,[26], [27], [28], [29], [30], [31], [32]].

### 2.2 Developmental disorders and stem cell differentiation

The association of TN with stem cell differentiation was reported for different types of stem cells. The potential role of TN in osteogenesis was suggested by showing the induction of TN expression at the mineralization stage of osteoblastic cells [12]. TN expression during osteogenesis was inhibited by transforming growth factor β1 and retinoic acid, regulators of bone formation [13,33]. TN expression is associated directly with bone formation in 3D osteospheroid cultures of human mesenchymal stem cells and bone marrow stromal cells [[34], [35], [36]]. In vivo studies confirmed the important role of TN in osteogenesis, which showed a spinal deformity named kyphosis and delayed fracture healing in TN knockout mice [14,37]. Although the molecular function of TN in osteogenesis remains to be elucidated, a recent study reported that the plasminogen activation system is closely related to osteogenesis and bone formation [38,39].

TN expression is also induced in developing muscles during embryogenesis, and TN protein was localized in myotendinous junctions [15]. In addition, TN expression was induced during skeletal muscle regeneration after a mouse injury. The association of TN with myogenesis was confirmed by a study that reported increases in TN expression during the myogenic differentiation of C2C12, mouse satellite cells, and embryonic stem cells. Recent studies consistently showed that TN is closely related to the protection of cardiac muscles and the regeneration of skeletal muscles [40,41]. A previous study showed that plasmin is also required for myogenesis, showing that inhibiting plasmin activity reduced the myogenic differentiation of C2C12 cells [41]. Another study reported that a plasminogen activator modulates myogenesis in mouse embryonic stem cells [42,43].

An analysis of gene expression in preadipocytes from different ages and depot origin suggested that TN is related to adipocyte differentiation, contributing to fat distribution and dysfunction [44]. Another study of proteomic analysis for human adipocyte culture medium identified TN as an adipokine, promoting adipogenesis and lipid synthesis [45]. TN was also identified as an adipogenic serum protein that enhances the adipocyte differentiation of 3T3-L1 mouse preadipocytes [16]. Recently, the mechanism of the adipogenic TN function was elucidated [18]. TN promoted the mitotic clonal expansion of growth-arrested 3T3-L1 cells, an essential differentiation step in the early phase of adipocyte differentiation. In addition, the TN-promoted mitotic clonal expansion was mediated via the ERK signaling pathway. The functional domain of TN for its adipogenic effect was identified to be the C-terminal region where the plasminogen binding site overlapped [17]. On the other hand, no direct relationship between TN and plasminogen activation was observed in adipocyte differentiation [17], even though the plasminogen activation system was suggested to be involved in the regulation of adipogenesis [42].

### 2.3 Neurological disorders

TN is also present in the cerebrospinal fluid (CSF), which may be produced by numerous neurons or selectively transported from the blood over the blood-brain barrier [46]. An analysis of CSF showed that the TN concentrations in CSF are decreased significantly in patients with various neurological disorders, including multiple sclerosis, compared to controls, suggesting the association of TN with neurological diseases (Fig. 1 and Table 1) [47]. However, in case of epilepsy the CSF TN concentration increased while serum and plasma TN decreased [48,49]. Proteomic analysis of CSF identified TN and showed that its expression was downregulated in patients with Parkinson’s disease (PD) [50]. TN expression was elevated significantly in PD patients after surgical therapy, whereas the expression levels decreased when the therapy ceased [51]. These results are supported further by in vivo studies of TN knockout mice showing the development of PD and enhancement of neuronal apoptosis [52,53]. Moreover, cohort studies reported that a missense variant of the TN gene (c.316G > A, p.S106G rs13963) is associated with longevity and neurologically healthy aging [54,55].

α-Synuclein is a major part of Lewy bodies in the brain of PD patients [56]. The aggregation of α-synuclein is mainly responsible for the degeneration of neurons and their cell-cell transmission, responsible for the progression of PD [57,58]. A recent study reported that exogenous TN alleviates synucleinopathies in a model cell line and reduces the cell-to-cell transmission of α-synuclein [59]. This study showed that TN-promoted degradation of α-synuclein by the activation of the plasminogen activation system and suggested the TN-plasmin-α-synuclein interaction, which explains the molecular function of TN to prevent PD progression.

### 2.4 Myocardial fibrosis and cardiovascular disease

A recent study reported that TN is a promising biomarker for heart failure associated with myocardial fibrosis [40]. The serum TN levels were reduced significantly, two-fold lower in patients than controls, and negatively correlated with circulating fibrosis markers. In contrast, TN in cardiac tissues was positively correlated with the fibrosis markers within the myocardium (Table 1). Similar results of the negative correlation of serum TN with heart failure were also obtained in the proteomic analysis of animal serum [60]. TN is also associated with cardiovascular disease and has been suggested as a serum/plasma biomarker by showing negative correlations of the TN serum/plasma level with acute myocardial infarction, atherosclerotic cardiovascular disease and coronary artery disease (Table 1) [[61], [62], [63], [64]]. Although its pathophysiological role is unclear, TN binding to angiostatin was suggested to inhibit the angiostatin activity of anti-angiogenesis [65], which might explain the association of TN with myocardial fibrosis and atherosclerotic cardiovascular disease.

### 2.5 Inflammation and sepsis

TN is produced by various types of lymphocytes, such as mast cells, neutrophils, monocytes, and macrophages [[66], [67], [68]]. The detection of TN in human lymph nodes suggested that TN is related to the immune system [69]. Other studies reported reduced TN levels in the serum and increased TN concentrations in the synovial fluid of patients with rheumatoid arthritis [70,71]. A recent study revealed the direct association of TN with sepsis and its molecular mechanism [19]. Serum TN was reduced significantly in septic patients and was depleted when the patient died of sepsis (Table 1). In addition, TN knockout mice are more susceptible to lethal sepsis and rescued by exogenous TN supplementation, indicating a beneficial role of TN in lethal sepsis. Importantly, TN specifically binds high mobility group box1 (HMGB1), enhancing the cellular uptake of HMGB1 that can cause hyperinflammation and immunosuppression in lethal sepsis [72,73].

### 2.6 Diabetes

The relationship between TN and diabetes is unclear, with different findings of elevated serum TN levels in type 1 diabetes [74] or inversely related to type 2 diabetes in other studies [75,76]. On the other hand, a recent study showed significant increases in the serum TN levels in type 2 diabetic humans and mice (Table 1) [77]. Serum TN was derived from adipose tissues, and TN expression in adipocytes was stimulated by high glucose via the p38 mitogen-activated protein kinase (MAPK)/thioredoxin-integrating protein (TXNIP)/thioredoxin (TX)/octamer-binding transcription factor 4 (OCT4) signaling pathway. In addition, TN knockout and inhibition of TN using neutralizing antibodies reduced the serum glucose with increased insulin levels in diabetic and high-fat-diet mice. Furthermore, exogenous TN treatment aggravated the hyperglycemia with reduced insulin secretion from pancreatic β cells. This study revealed the specific TN binding to pancreatic β cells and blocking of a subtype of L-type calcium channels (LTCC), i.e., voltage-gated calcium 1 (Cav 1.3) responsible for inhibiting insulin secretion, suggesting that TN would be a promising therapeutic target for type 2 diabetes. Another recent study on type 2 diabetes extensively studied the protein profile of patient’s serum samples using a label-free LC-MS/MS technique, which identified the downregulation of TN (CLEC3B) protein in type 2 diabetes patients [78].

## 3 Molecular structure and conserved domains

The human TN gene, Clec3B (C-type lectin domain family 3B), was identified in the early 1990s, which consisted of three exons with 606 base pairs for 202 amino acids [79,80]. A gene for TN was found in most vertebrates, and the protein is highly conserved in mammals, showing ≥70 % identities in amino acid sequences. N-terminal 21 amino acids were identified as a signal peptide for protein secretion, absent in TN purified from human plasma [81]. The secretion signal peptide was followed by a lysine-rich region (1–15) characterized as a heparin-binding domain [82,83]. Mutation analysis showed that lysine residues directly contribute to TN binding with heparin, even though its heparin-binding affinity was estimated to be lower than other heparin-binding proteins [82]. Following region (16–49) was identified as a long α-helical domain involved in the interaction of TN monomers stabilizing an oligomeric structure [84,85]. The C-terminal region of 132 amino acids (50–181) is homologous to the carbohydrate recognition domains (CRD) of the calcium-dependent lectin superfamily, which classified TN into the superfamily of C-type lectins [86,87]. On the other hand, carbohydrate-binding in CRD has rarely been identified. Instead, the C-terminal region of TN is the binding site for plasminogen, interacting with the kringle-4 domain [88,89].

**Fig. 2:**
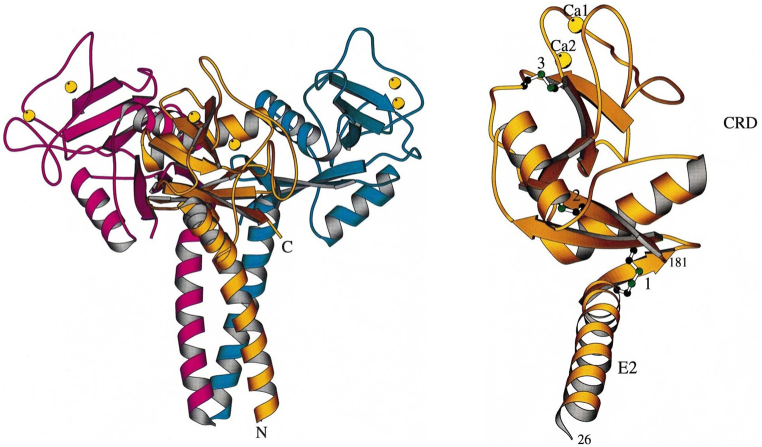
Structure of TN. The trimeric structure of TN on left, and the monomeric structure on right. TN consists of a long a-helical domain (E2) and a carbohydrate recognition domain (CRD). The yellow spheres denote two calcium ions, and the three-disulfide bridges are represented as ball and stick (figures adapted from Nielsen et al., 1997). (For interpretation of the references to color in this figure legend, the reader is referred to the Web version of this article.)

The crystal structure of TN revealed a homotrimeric structure of the protein, stabilized by the triple-coiled α-helix of oligomerization domains (Fig. 2) [85]. The homotrimeric structure of TN was stabilized by intramolecular non-covalent interactions and disulfide bridges between the CRD and the oligomerization domain. In addition, two calcium-binding sites are located in each CRD of TN (Fig. 2A). Calcium-dependent interactions between TN and sulfated polysaccharides, apolipoprotein, and fibrin were reported, even though the binding sites in TN have not been identified [83,90,91]. Other studies reported that the Plg binding to TN is inhibited in the presence of calcium [88]. In addition, the structure of the monomeric CRD domain assumed that the Plg binding site is less flexible and accessible for Plg binding than calcium-free apo-TN3, which might explain the inhibition of calcium-dependent inhibition of Plg binding to TN [92]. These studies suggest the calcium-dependent regulation of the ligand specificity of TN or (and) the protein function. On the other hand, the TN activity of enhancing Plg activation was barely changed in the presence of calcium at millimolar concentrations [17]. The calcium-dependent binding of TN to a ligand appears to be dependent on the assay conditions, and its functional and physiological relevance needs to be elucidated.

## 4 TN-binding proteins

### 4.1 Plasminogen

TN was first discovered as a plasminogen-binding protein [1]. Plasminogen is a zymogen that is activated by plasminogen activator-catalyzed cleavage to form the active form of plasmin. Plasmin accepts diverse substrate targets with broad specificity. Therefore, plasminogen and plasmin play important pathological roles in cancers, inflammation, and tissue regeneration [93]. Plasminogen contains seven domains: an N-terminal pan-apple domain, kringle domains 1–5, and a serine protease domain [94]. TN binds the kringle 4 domain of plasminogen with a binding affinity of *K*_d_ = 20–50 μM (Table 2) [88,89]. Although the structure of the TN-plasminogen complex is unavailable, the TN binding to the kringle 4 domain might cause conformational changes of plasminogen from its closed form to its favorable open form for the activation of plasmin [94].

### 4.2 Fibrin

Fibrin is a key protein involved in the clotting cascade formed by the thrombin-catalyzed polymerization of fibrinogen [95] (Table 2). TN was assumed to bind to fibrin by finding the presence of TN in clot lysates [91]. TN binding to fibrin appears to be dependent on calcium chloride but independent of plasminogen. Platelet-released TN mainly binds fibrin in blood coagulation, suggesting the involvement of TN in a specific local thrombus-directed mechanism [63,91]. The amount of TN bound to approximately 25 nmol/L, and the binding is in the same order of magnitude as Plg-TN binding (Table 2) [91]. On the other hand, there is no direct evidence of the molecular binding between TN and fibrin.

### 4.3 Apolipoprotein A

Apolipoprotein A (ApoA) is a major protein component of the high-density lipoprotein and plays an essential role in reverse cholesterol transport [96]. The protein has a multifunctional role in apoptosis, inflammation, immunity, microbial infections, and lowering the risk of cardiovascular diseases. The solution structure of APOA was studied using a small-angle neutron scattering technique and mass spectrometry (hydrogen/deuterium exchange) [97] (Table 2). It has been reported to have a therapeutic role in Alzheimer’s disease [98]. Kluft et al. assumed the functional analogy between ApoA and plasminogen regarding binding to TN, as both proteins contain homologous kringle 4 structures [90]. They revealed the binding of ApoA to TN with an estimated *K*_d_ = 0.013 μM (Table 2). On the other hand, no binding was observed between ApoA and fibrin-bound TN and fibrin, indicating no function of ApoA in the clotting system [90].

### 4.4 Hepatocyte growth factor and tissue plasminogen activator

Hepatocyte growth factor (HGF) is a glycoprotein of α and β heterodimer linked covalently by disulfide bonds. The α chain of HGF contains four kringle domains and an N-terminal heparin-binding domain. Uchikawa et al., described the overall domain structure of HGF while studying the binding of C-MET receptor (belongs to tyrosine kinase family) to HGF [99] (Table 2). HGF also functions as a mitogen for various types of cells, including melanocytes, keratinocytes, hepatocytes, and epithelial cells. In 2003, Westergaard et al. reported TN-binding molecules HGF and tPA. TN binding with HGF and tPA was initially confirmed by ligand blot analysis using plasminogen as a positive control. A solid-phase binding assay (ELISA) validated the affinities between TN and HGF/tPA. The results revealed the concentration-dependent binding for HGF and tPA with the calculated *K*_d_ values of 0.49 μM and 0.28 μM, respectively (Table 2) [100]. The apparent binding affinities for HGF and tPA were determined to be similar to the affinity for plasminogen. They assumed that the binding domain of TN would be the same for different ligands. Moreover, they reported that TN does not bind other kringle domain-containing proteins, urokinase-plasminogen activator (uPA), and macrophage-stimulating protein (MSP).

### 4.5 High mobility group box-1 (HMGB-1)

HMGB1 was first discovered in 1973 as a ubiquitous non-histone nuclear protein consisting of a large number of acidic and basic amino acids [101]. HMGB1 is involved in regulating the key processes in transcription. In addition, it regulates innate immune response and inflammation when released extracellularly from various cells and further acts on specific cell surface receptors [102,103]. Therefore, HMGB1 plays a pivotal role in various infectious diseases, cancer progression, neurodegenerative disorders, autoimmune diseases, and inflammatory disorders, including sepsis [104]. The structure of this 30 kDa protein consists of three functional domains - positively charged A box and B box, which functions as the DNA binding domain and one negatively charged acidic tail, and the N-terminus composed of heparin-binding sequence [[105], [106], [107]] (Table 2). Recently, Chen et al. reported the specific binding of TN to HMGB1 with a *K*_d_ = 1.21–2.88 nM and their involvement in lethal sepsis (Table 2) [19]. The cellular uptake of HMGB1 is facilitated by the formation of TN/HGMB1 complex, which induces macrophage pyroptosis that can cause hyperinflammation and immunosuppression in sepsis. Moreover, a monoclonal antibody specific for TN inhibits the TN/HMGB1 interaction, preventing the cellular uptake of HMGB1 and rescuing animals from lethal sepsis. This study strongly suggests that TN can be a therapeutic target in sepsis and other diseases by inhibiting complex formation between TN and its binding effector molecules.

## 5 Other TN-binding molecules

### 5.1 Heparin

Heparin is a heterogenous polyanionic carbohydrate and plays a significant role in various biological functions, such as anticoagulant and anti-inflammatory activity, development of nerve cells, tumor development, invasion and angiogenesis, and cell proliferation of smooth muscle cells [108]. TN binds with sulfated polysaccharides heparin in the N-terminal region, where five lysine residues are involved in heparin binding, as revealed by mutational analysis [82,83]. The binding affinity of heparin–sepharose with recombinant TN was determined by semi-quantitative affinity chromatography, and the *K*_d_ value was 65 μM (Table 2) [82]. The heparin-binding affinity of TN is relatively low compared to other heparin-binding proteins. The physiological function of TN might be related to heparin binding, but no direct association with the disease has been reported.

### 5.2 Epigallocatechin gallate (EGCG)

**Fig. 3:**
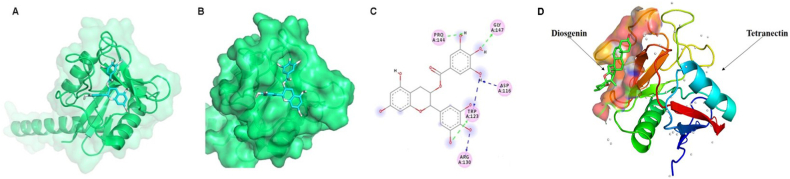
Model structures of TN-EGCG and TN-diosgenin complexes. **A**, ribbon representation of the TN structure bound with EGCG in a stick representation. **B**, surface representation of TN-EGCG complex. **C**, amino acid residues directly interacting with EGCG (figures adapted from Iram et al., 2022). **D**, ribbon representation of the TN structure bound with diosgenin in a stick representation (the figure adapted from Amin et al., 2023).

EGCG is the first natural compound that showed binding with TN [20]. EGCG is the most abundant active catechin (polyphenol) in green tea. EGCG has been established as a well-known anti-oxidant, anti-inflammatory, anti-fibrotic, and tissue-protective compound because of its interactions with various intracellular signaling pathways, cell surface receptors, and nuclear transcription factors. These protective properties and interactions made EGCG an essential natural compound that might treat several diseases, such as cancer and neurological, respiratory, cardiovascular, and metabolic disorders [109]. The chemical structure of EGCG consists of a bicyclic (benzopyran) ring attached to gallate and a trihydroxy benzene ring, which binds to TN in the carbohydrate recognition domain (Fig. 3A–C). The binding affinity between TN-EGCG complex was determined with fluorescence quenching assay and isothermal titration calorimetry, and the *K*_d_ value was found to be in the range of 3–6 μM (Table 2). A previous study reported that the *K*_d_ values for the TN plasminogen complex were 0.3–0.5 μM [88], suggesting that EGCG competes with the plasminogen to bind TN, inhibiting the TN activity of enhancing plasminogen activation. They revealed the cytotoxic effect of EGCG on mouse colon cancer cells (CT-26) partly mediated by its inhibitory effect on TN.

### 5.3 Diosgenin

Another natural compound which showed binding to TN is diosgenin (Table 2). Diosgenin, a steroidal sapogenin, is present in fenugreek seeds as a major bioactive compound and exhibits potent ant-proliferative, anti-oxidant, and anti-inflammatory, activities [110]. Diosgenin is also known to inhibit the proliferation of cancerous cells such as breast, osteosarcoma, and hepatocellular [[111], [112], [113]]. A recent in silico study identified diosgenin to bind TN in the carbohydrate recognition domain, and likely to form a stable complex [114] (Fig. 3D). In addition, diosgenin significantly suppressed the proliferation and the migration of breast cancer cells. This study suggested diosgenin as an alternative treatment to reduce breast cancer metastasis, although the binding of diosgenin to TN and its TN inhibition mechanism are not yet completely elucidated on molecular and cellular levels.

## 6 Conclusion and perspectives

Over the past 36 years, research has suggested the critical role of TN in various human diseases. Most studies reported that TN functions by binding target molecules and regulating their function in related diseases (Table 2). TN-binding targets are diverse, but they are mostly proteins and appeared to be specific for TN binding. Plasminogen and HMGB1 are the most well-studied TN-binding proteins, which mediate the association of TN with cancer development and lethal sepsis, respectively. TN regulates the activation of plasminogen and the transport of HMGB1 into cells. TN binds the target proteins in its conserved C-terminal region, although TN binding sites on target proteins are vaguely known. Interestingly, recent studies have shown that an antibody specifically binding the C-terminal region of TN inhibits the interaction of TN with HMGB1 [19]. More importantly, the TN-targeted antibody effectively decreased lethality in sepsis mouse model. We also recently identified the natural compound EGCG to bind the C-terminal region of TN, which effectively suppresses cancer cell proliferation by inhibiting the interaction between TN and plasminogen [20]. The therapeutic effect of EGCG has been reported for TN-associated diseases, although the molecular mechanism is not completely elucidated [96]. Moreover, the other natural compound, diosgenin, was identified as an effective TN inhibitor suppressing plasminogen activation and breast cancer metastasis [114]. These studies strongly suggest that TN is a versatile therapeutic target, not only for sepsis and cancer, but also for other TN-associated diseases. Although some TN binding target proteins have been identified, only HMGB1 and plasminogen are relatively well elucidated to mediate sepsis and cancers, respectively, which lead to the development of potential TN-targeted drugs. The role of TN appeared to be heterogenous in different diseases due to different TN binding targets to mediate diseases. Therefore, future studies should be focused on identification of TN-binding proteins specific to mediate different diseases. In addition, elucidation of their molecular and cellular functions would expand the development of TN-targeted drugs for various TN-associated diseases.

## Data availability statement

No data was used for the research described in the article**.**

## CRediT authorship contribution statement

**Sana Iram:** Writing – review & editing, Writing – original draft, Visualization, Investigation, Formal analysis. **Safikur Rahman:** Writing – review & editing, Writing – original draft, Visualization, Validation, Formal analysis. **Inho Choi:** Writing – review & editing, Validation, Supervision, Project administration, Funding acquisition. **Jihoe Kim:** Writing – review & editing, Writing – original draft, Visualization, Validation, Supervision, Software, Resources, Project administration, Methodology, Investigation, Funding acquisition, Formal analysis, Data curation, Conceptualization.

## Declaration of competing interest

The authors declare that they have no known competing financial interests or personal relationships that could have appeared to influence the work reported in this paper.
